# Prospectus of a Bi-Monthly Record of Obstetric Medicine and Surgery, and of the Diseases of Women and Children

**Published:** 1848-01

**Authors:** 

**Affiliations:** Manchester


### RECORD OF MEDICAL SCIENCE.

*Prospectus of a Bi-Monthly Record of Obstetric Medicine and Surgery, and of the Diseases of Women and Children.* Edited by Dr. Clay, Manchester.

It has long been evident to practical medical men that Obstetrics have never been duly cared for, or encouraged by the medical press of this country, whilst at the same time it must be acknowledged that the articles produced from such resources yield to no other in interest, importance, or utility.

The Editor therefore proposes to fill up this hiatus by establishing a periodical entirely devoted to Obstetric Medicine and Surgery with the Diseases of Women and Children. And though it will be a new feature in English Medical literature to treat upon one branch of Medical Science exclusively—yet it is hoped greater justice will be rendered to the department selected by this means than could possibly be obtained by any other. A similar plan has already been adopted on the continent and succeeded admirably, it is therefore presumed an effort at home will not prove abortive.

From the cordial promises of support and assistance from many eminent Accoucheurs, British and Foreign, the Editor feels no doubt in asserting that the contemplated periodical will contain papers of the highest value and interest to practitioners, and that the Journal itself will yield to none in respectability, talents of its contributors, and the strict professional character of its arangement. A number of very valuable papers are already promised, and will be inserted in the early numbers.

As it is the intention of the Editor to conduct the Journal upon new principles, a brief outline of the plan will be necessary.

*Plan.*—Every Communication, Book, or even Advertisement, that is based on Quackery, or couched in unprofessional language, will be rejected. And although such are often a source of considerable profit to an Editor, he is determined not to allow even the cover of this publication for such a purpose; trusting solely to the extensive support of regular practitioners.

Medical Politics will have no place in the pages of this periodical.

Leading Articles, (excepting the Editorial Address in the first number) will at all times be very short, and generally confined to practical information.

For Anonymous Reviews, the Editor intends to substitute an entirely new plan, viz: The author to furnish the work to the Editor, accompanied with a faithful abstract of all that is material in the work, giving a clear idea of the nature of the same. The Editor reserving his right of comment.

A Monthly Retrospect of Obstetric Information, selected with care from all the British and Foreign Journals.

Occasionally will be reprinted, and paged separately, so as to bind up as independent works—Translations of extremely scarce and valuable Monographs, from the Latin, French, German, Dutch, and other languages, Ancient and Modern—Reprints of very scarce English Monographs—and the printing of rare and valuable Manuscripts; of these many are now in possession of the Editor, who will also esteem it a favour to receive such curiosities from his supporters for publication, after which the originals will be most punctually returned.

The ANCIENT MONOGRAPHS will be arranged in epochs:—

*First.* Commencing with ***Hippocrates***, about 450 B. C., including all in reference to Obstetrics, from the best writers, to the year A.D. 1000, ending with *Nonnus.*

*Second.* Commencing with ***Avicenna***, including the writers to A. D. 1500, ending with ***Rhodion.***

*Third.* Commencing with ***Bonaciolus***, including the writers to A. D. 1650, ending with ***Riviere.***

*Fourth.* Commencing with *Primrose*, in 1650, including authors up to 1760.

*Fifth.* Modern Scarce Monographs.

Sixth Division will be the printing of rare Manuscripts.

The Editor has also been for some years engaged in compiling an Encyclopædia of the past and present state of Obstetric Medicine and Surgery, including the diseases of Women and Children, (at least a thousand articles of which are already prepared) and should he be liberally supported, he proposes to publish a few pages in each number of the Journal so as to bind up as a separate work, forming a complete Encyclopædia of General Reference to the Student and Practitioner, bringing down the science of Obstetrics from the earliest period of history to the present time. Illustrated by some hundreds of wood-cuts, &c.

The Editor having in his possession a most extensive library of Obstetric authorities, ancient and modern, consisting of works in most European Languages, collected at great expense, he proposes to use it for the benefit of his readers in answers to Practical, Theoretical, Historical, Biographical, and Bibliographical questions.

The usual Journal Information will not be neglected; Notices of new publications; Appointments; Obituary; Biography of past and Sketches of living Authors; Questions answered. Excerpts from rare works, selected as curiosities, or for their practical value. Notices to Correspondents. A list of contributors and subscribers will occasionally be published, &c., &c.

Such are the principles on which it is proposed to conduct this periodical, the pages of which will at all times be open to encourage professional improvements; towards their advancement any communication connected with the objects of the journal will be thankfully received.

